# Staphylococcal enterotoxins modulate the effector CD4^+^ T cell response by reshaping the gene expression profile in adults with atopic dermatitis

**DOI:** 10.1038/s41598-019-49421-5

**Published:** 2019-09-11

**Authors:** Raquel Leao Orfali, Fabio Seiti Yamada Yoshikawa, Luanda Mara da Silva Oliveira, Natalli Zanete Pereira, Josenilson Feitosa de Lima, Yasmim Álefe Leuzzi Ramos, Alberto José da Silva Duarte, Maria Notomi Sato, Valeria Aoki

**Affiliations:** Department of Dermatology, Laboratory of Dermatology and Immunodeficiencies (LIM-56), Faculdade de Medicina FMUSP, Universidade de Sao Paulo, Sao Paulo, SP Brazil

**Keywords:** Lymphocyte activation, Inflammation

## Abstract

*Staphylococcus aureus* colonizes the skin of atopic dermatitis (AD) individuals, but the impact of its enterotoxins on the chronic activation of CD4^+^ T cells demands further analysis. We aimed to analyze the CD4^+^ T cell anergy profile and their phenotypic and functional features through differential expression of cellular activation markers, cytokine production and response to staphylococcal enterotoxin A (SEA). A panel of 84 genes relevant to T cell anergy was assessed by PCR array in FACS-sorted CD4^+^ T cells, and the most prominent genes were validated by RT-qPCR. We evaluated frequencies of circulating CD4^+^ T cells secreting single or multiple (polyfunctional) cytokines (IL-17A, IL-22, TNF, IFN-γ, and MIP-1β) and expression of activation marker CD38 in response to SEA stimulation by flow cytometry. Our main findings indicated upregulation of anergy-related genes (*EGR2* and *IL13)* promoted by SEA in AD patients, associated to a compromised polyfunctional response particularly in CD4^+^CD38^+^ T cells in response to antigen stimulation. The pathogenic role of staphylococcal enterotoxins in adult AD can be explained by their ability to downmodulate the activated effector T cell response, altering gene expression profile such as *EGR2* induction, and may contribute to negative regulation of polyfunctional CD4^+^ T cells in these patients.

## Introduction

Atopic dermatitis (AD) is a widespread, chronic, inflammatory, immune-mediated and pruritic skin disease^1^. Interactions among susceptibility genes encrypting skin barrier molecules^2^, inflammatory response elements, environmental factors and infectious agents (mainly *Staphylococcus aureus* and herpes virus), together with the distorted immunologic status of the host, are critical elements in AD pathophysiology^3,4^.

Classically, AD immune pathogenesis is described as an imbalance of the human T helper (Th) cell subsets Th1 and Th2^5^. However, due to the characterization of the new subsets Th17 and Th22^6^, novel inflammatory components in AD require better evaluation^3,5,7^. Th17-derived IL-17 is capable to orchestrate local tissue inflammation through upregulation of pro-inflammatory cytokines and chemokines, comprising IL-6, TNF-α, IL-1β, CXCL1, CCL2, CXCL2, CCL7 and CCL20.^7^ In collaboration with IL-17, IL-22 induce antimicrobial peptide production and initiates an acute phase response^8,9^. It has been hypothesized that elevated IL-22 and diminished IL-17 expression predominate in the chronic phase of AD; therefore, the early hypothesis describing AD as a Th2-driven disorder should be modified to accommodate the Th22/Tc22 subsets associated with epidermal changes^10–12^.

*Staphylococcus aureus* (*S*. *aureus*) colonizes the skin in approximately 30–50% of healthy adults, but it is consistently detected in 10–20% of colonized adults^13^. In more than 90% of AD patients, the skin shows an increased *S*. *aureus* colonization^14^, with well-known occurrence of antibiotic-resistant strains (methicillin-resistant *Staphylococcus aureus*, MRSA)^15^. Decreases in the cutaneous barrier function (as diminished expression of filaggrin and human β-defensin 3) and release of Th2-associated cytokines, favor *S*. *aureus* skin penetration in AD subjects^16–19^. Staphylococcal exotoxins can promote the release of proinflammatory mediators from cutaneous cells and lead to subsequent pruritus and scratching, binding to specific IgE on the surface of Langerhans cells, facilitating allergen presentation, and activation of specific T cells^20^. Cutaneous *S*. *aureus* is also accomplished of inducing differentiation of Gr1^+^CD11b^+^ myeloid-derived suppressor cells, leading to immune suppression of T cell activation in skin, and decreased numbers of splenic CD4^+^ and CD8^+^ T cells in mouse models^21^.

Effector memory T cells driven against antigens derived from cutaneous pathogens, such as *S*. *aureus*, are essential for providing rapid defense against infections^22^. The hallmark functions of CD4^+^ T cells include cytokine production and optimization and maintenance of CD8 T cell memory^23^. Polyfunctional T cells comprise distinct functional subsets of effector T cells with the ability to produce and release different combinations of cytokines during the course of the immune response^24^. Compared with subsets that secrete single cytokines, polyfunctional T cells provide more effective, long-lasting protection and enhance other effector functions^9,25–27^, making them an interesting target for vaccine and immunotherapies designs that are dependent on cellular responses^28–31^. However, whether polyfunctional T cells could be involved in AD have never been assessed.

In this paper, we present a novel approach to study the status of CD4^+^ T cells in AD based on their polyfunctional profile. By analyzing the phenotypic features of CD4^+^ T cells exposed to staphylococcal enterotoxins, we observed impairment in the ability of chronically activated cells from AD patients to develop a polyfunctional response. These superantigens seem to reshape the genetic program of those cells into an anergic profile that may contribute to the chronicity of AD.

## Results

### Staphylococcal enterotoxin triggers a broad tolerogenic genetic program

Considering the chronic nature of *S*. *aureus* colonization and the known defects in effector immune response in AD patients, we propose that staphylococcal enterotoxins can drive CD4^+^ T cell from AD patients to a tolerogenic profile. In order to test this hypothesis, we performed a PCR array for 84 key genes related to T-cell anergy and immune tolerance in cell-sorted CD4^+^ T cells from PBMC upon SEA stimulation.

**Figure 1:**
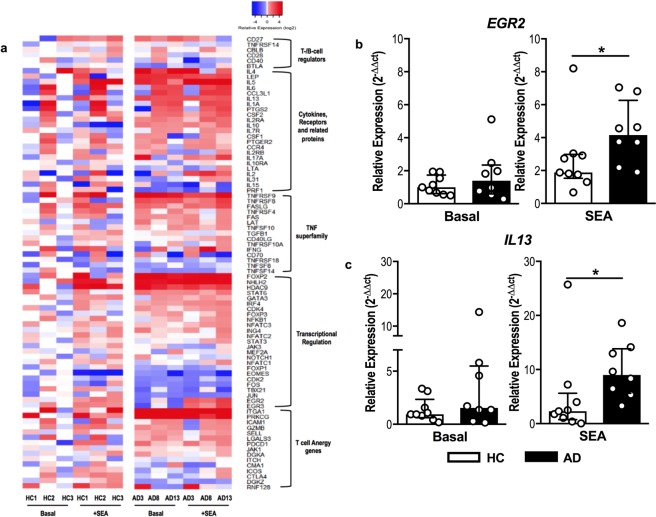
Staphylococcal enterotoxins drive CD4^+^ T cells to an anergic profile in AD. (**a**) Heatmap of the relative expression values of 84 key genes related to T-cell anergy in FACS-sorted CD4^+^ T cells analyzed by PCR array. The genes were classified according to functional criteria expressed on a thermal scale for HC (n = 3) and AD patients (n = 3) at baseline and after SEA stimulation. Expression of upregulated genes *EGR2* (**b**) and *IL13* (**c**) was confirmed by RT-qPCR (n = 8–9). Bars represent median with interquartile range. *p < 0.05.

As illustrated in the heatmap (Fig. 1a) and the volcano plots (Supplementary Fig. S1), a multitude of anergy related genes are upregulated in AD patients, including the classical AD associated ones *IL4* and *IL13*; the last one has been also validated by conventional qPCR (Fig. 1c). It should be highlighted that most of the screened genes have never been linked to AD, which may open new venues for investigation. However, a previous genome-wide association study by Hirota *et al*.^32^ suggested that the early growth response (EGR) *EGR2*, a negative regulator of T cell activation^33^, could be a susceptibility locus for AD. Notably, we found significant expression of *EGR2* in AD patients, which was validated by conventional qPCR (Fig. 1b). These findings indicate that SEA is able to arrest the CD4^+^ T cell of AD patients to an anergic program, which may account greatly for their impaired immune response.

### Staphylococcal enterotoxins compromises CD4^+^ T cell polyfunctional profile in AD

In previous studies, our group demonstrated a diminished peripheral blood mononuclear cells (PBMCs) proliferation response to staphylococcal enterotoxin A (SEA) and other antigens and mitogens (Tetanic toxoid, TT; *Candida albicans* membrane antigen, CMA; and phytohemagglutinin, PHA), suggestive of a defective immune profile in adults with AD^34^. In line with the anergic response observed above, the CD4^+^ T cell response seems to be affected in AD.

**Figure 2:**
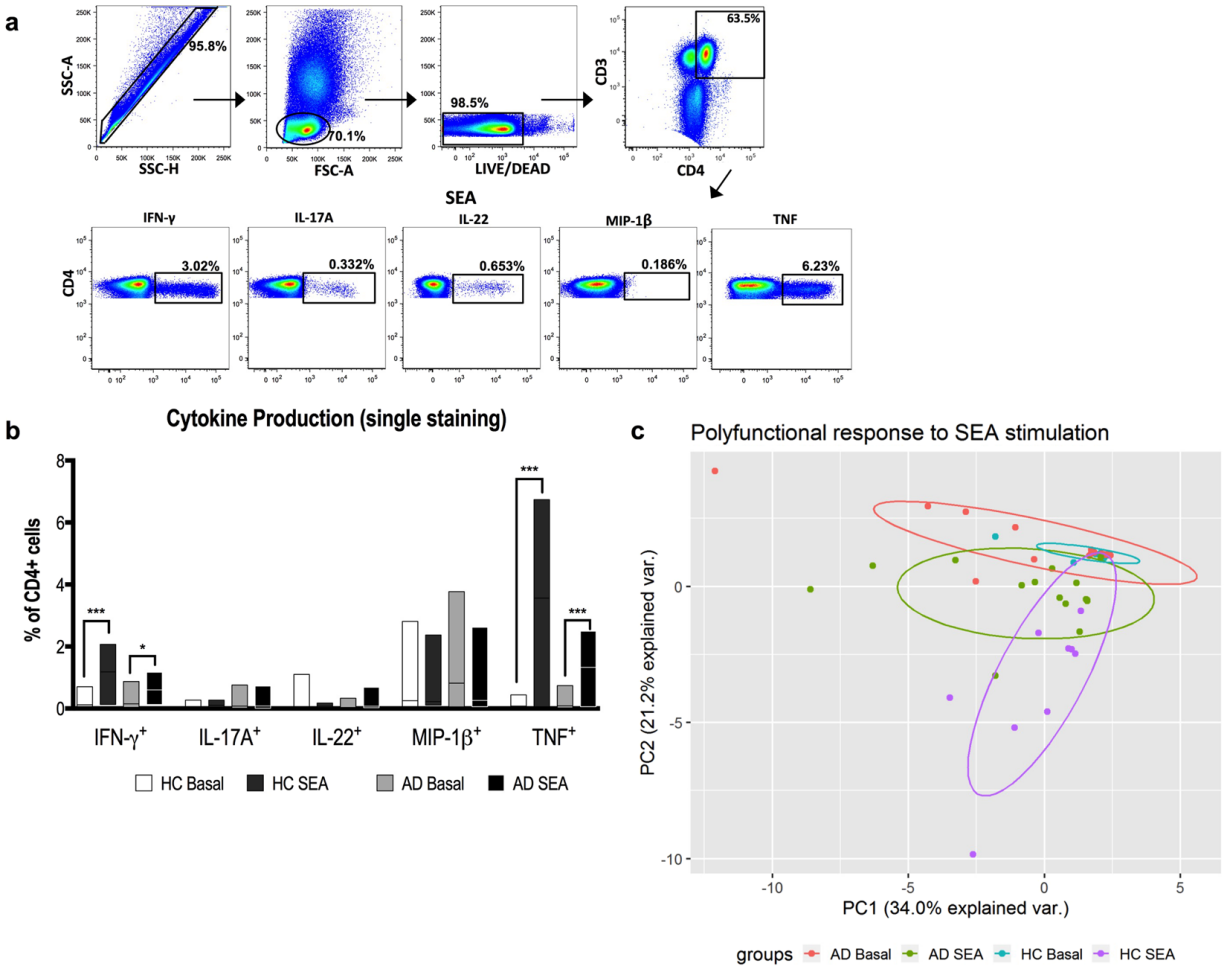
Impaired T cell cytokine secretion induced by SEA in AD. (**a**) Representative gating strategy for selection of CD3^+^CD4^+^ T cell population. Each subsequent panel shows only the population of interest producing each studied cytokine after SEA stimulation. (**b**) Frequencies of CD4^+^ T cells individually stained for IFN-γ, IL-17A, IL-22, MIP-1β or TNF after SEA stimulation from HC (n = 10) and AD patients (n = 15). Lines represent median with interquartile range. *p < 0.05, **p < 0.01, ***p < 0.001. (**c**) PCA of the CD4^+^ T cells polyfunctional profile. Boolean gating was used to calculate the proportions of polyfunctional T cells after SEA stimulation and frequency values were considered for analysis. Each dot represents one subject. *PC*, principal component.

Considering the relevance of Th1, Th2, Th22 and Th17 axes to AD immunopathogenesis, first, we evaluated the influence of staphylococcal enterotoxins (SEA and SEB) on CD4^+^ T cells producing IFN-γ, IL-17A, IL-22, MIP-1β and TNF according to the gating strategy shown in Fig. 2a. Curiously, when we considered the phenotype based on single staining for each cytokine (Fig. 2b), AD-derived CD4^+^ T cells were as efficient as health controls in IFN-γ and TNF production, apparently arguing against our depressed immune response hypothesis.

However, instead of a single cytokine response, a polyfunctional phenotype should be more representative of a cell activity and it has been associated with beneficial immune responses^28^. Therefore, we next aimed to analyze the CD4^+^ T cells ability to simultaneously produce the above cytokines upon SEA stimulation. Due to the great amount of data generated in polyfunctional analysis (Supplementary Fig. S2), a PCA was applied to understand the groups’ dynamics in response to SEA.

As shown in Fig. 2c, we observed that HC display a remarkable polyfunctional response, moving from a condensed basal cluster toward a dispersed distribution pattern after SEA addition. Strikingly, AD patients showed a compromised profile upon the same stimulation, seen by the similar dispersion and partial overlay of both clusters (basal and SEA stimulated), which suggests that the basal CD4^+^ cell response is already altered in AD and SEA exerts a mild effect in promoting cytokine production. Thus, this impaired cytokine profile in AD patients to staphylococcal enterotoxins may reflect an unresponsive status of their CD4^+^ T cells.

### CD4^+^ T cells in AD patients are chronically activated but show reduced polyfunctional response to SEA stimulation

The persistent skin colonization by *S*. *aureus* may drive a chronic activation of the immune system. This activation status can be monitored in a T cell through changes in phenotypic markers, such as CD38, a chronic activation marker already reported in CLA^+^CD4^+^/CD8^+^ T cells from individuals with extrinsic AD^35^. CD38 expression is also found in thymocytes, B lymphocytes, circulating monocytes, natural killer cells and granulocytes^36^, and reflects a T cell activation in response to microbial infection or vaccination^37^. Thus, in order to filter the above results, we next investigated whether a differential polyfunctional profile could be detected based on the CD38 expression.

**Figure 3:**
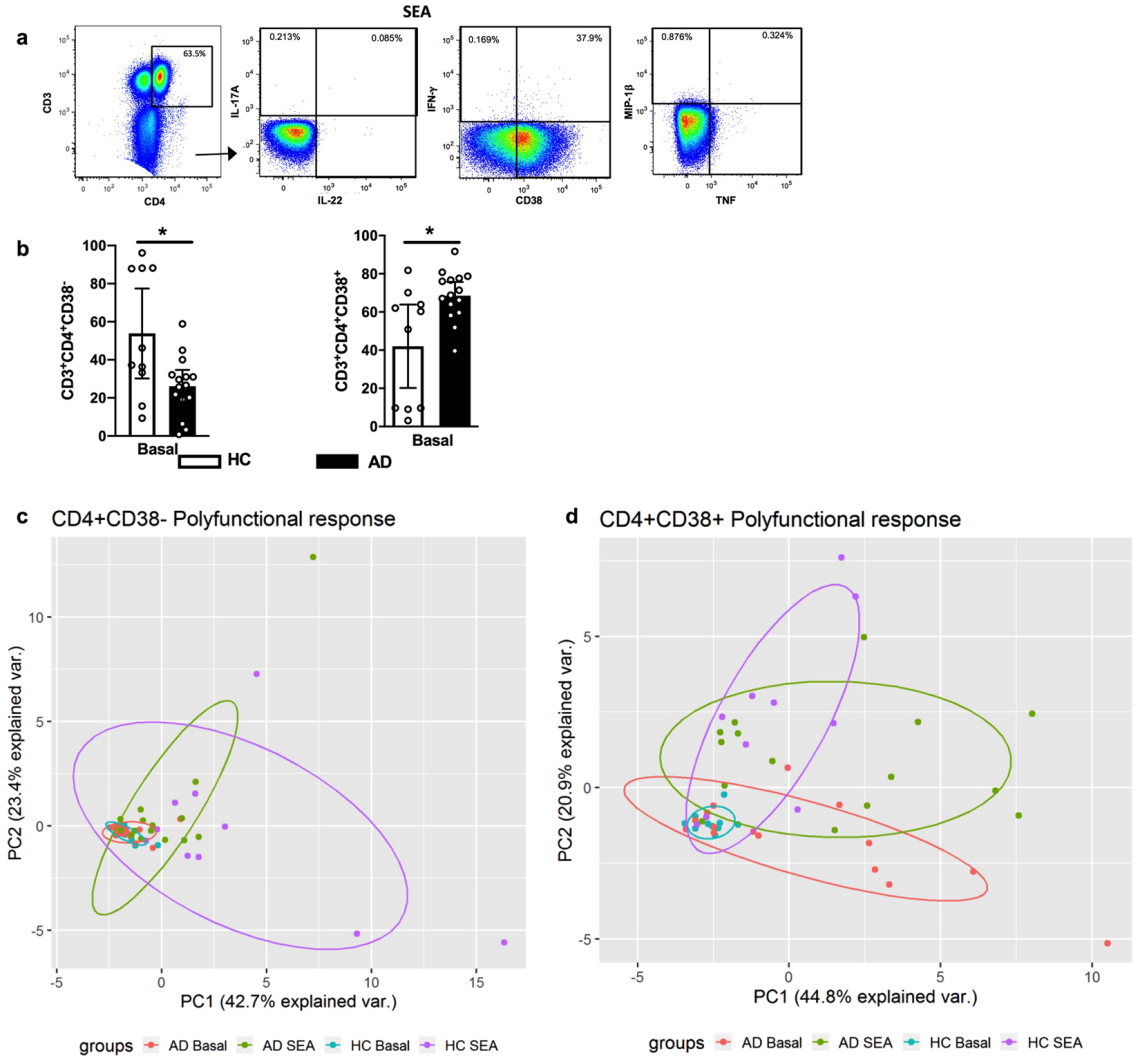
The high frequency of *ex vivo* CD4^+^CD38^+^ polyfunctional T cells is associated with a dysfunctional response to SEA in AD patients. (**a**) Representative gating strategy for analysis of cytokines and CD38 in CD4^+^ T cells. (**b**) Frequencies of CD4^+^CD38^+^/CD4^+^CD38^−^ T cells in HC (n = 10) and AD patients (n = 15) in unstimulated condition. Bars represent median values. *p < 0.05. (**c**,**d**) PCA of the polyfunctional profile of CD4^+^CD38^+^ and CD4^+^CD38^−^ T cells. Boolean gating was used to calculate the proportions of polyfunctional T cells after SEA stimulation and frequency values were considered for analysis. Each dot represents one subject. *PC*, principal component.

Initially, we evaluated the marker expression in CD4^+^ T cells, according to the gating strategy shown in Fig. 3a, and we detected an increased frequency in AD patients in unstimulated condition (Fig. 3b), corroborating the chronic activation profile classically seen in those patients.

Curiously, in CD4^+^CD38^−^ T cells, AD lymphocytes responded to SEA similarly to HC counterparts (Figs 3c, S3). However, when focusing on the CD4^+^CD38^+^ population, we could recapitulate the deficient response of total CD4^+^ cells in AD (Figs 3d, S4), reproducing the mild changes in cluster dynamics between basal and SEA stimulation in AD group. Therefore, the total CD4^+^ T cell response is in fact heterogeneous and the prevalent anergy profile seems to be restricted to CD38^+^ subsets. Thus, our data reinforces the need to understand the CD4^+^ T cell response at the subpopulation level. Together, our findings indicate that *ex vivo* CD4^+^CD38^+^ T cells are chronically activated in AD patients and show compromised polyfunctional response to staphylococcal antigens.

## Discussion

AD is a complex disease that mobilizes multiple branches of the immune system. Our study, focusing on AD in adults, demonstrated the enhanced expression of *EGR2* and *IL13* genes upon SEA stimulation and the presence of *ex vivo* chronically activated polyfunctional CD4^+^CD38^+^ T cells, indicating a negative regulatory pathway modulated by staphylococcal enterotoxins.

The current group of atopic patients had AD with long-term duration, with a mean length of disease of 21.2 years, increased IL-17 expression in skin lesions and higher IL-17 serum levels compared to controls^38^. They also exhibit IL-22-expressing CD4/CD8 T cells in their cutaneous lesions, show imbalanced circulating Th22 cells, and display Tc22 cell induction after staphylococcal enterotoxin stimulation, with enhanced expression of *IL4* and *IL22* in AD skin^11^; altogether emphasizing their Th2/T22-biased response^11^. In addition, adult patients with AD have defective antigen-specific and polyclonal proliferative responses^34^, suggesting an immunosuppressed profile in their CD4^+^ T cell compartment. Although healthy individuals can be exposed to staphylococcal superantigens, we hypothesize that AD patients may show an altered response to antigenic stimulation^34,39^.

Previous works addressing the role of staphylococcal exotoxins in AD, including both children and adults, showed an immunostimulatory rather than an immunosuppressive effect^7,40,41^, albeit those contradictory responses could be due to the nature of the antigen utilized and the patients’ age.

By broadly screening the expression of genes related to T cell anergy, we observed a marked induction of *EGR2* expression by SEA stimulation. The EGR genes *EGR2* and *EGR3* were previously associated with anergy and negative regulation of T cell function^33^, with *EGR2* also described as a susceptibility locus for AD at genome-wide significance in the Japanese population, and reported as a candidate gene associated with regulatory T cells^32,42^. Although its expression was demonstrated in murine regulatory T cells (CD4^+^CD25^–^LAG3^+^)^43^ our present data brings new human experimental evidence that reinforces the possible link between this gene and AD.

Additionally, our current data on T lymphocyte polyfunctionality adds a new layer of complexity to the biology of CD4^+^ T cells in AD pathogenesis, as we detected a downregulatory effect by staphylococcal enterotoxins on cytokine secretion in AD subjects. Interestingly, this response is remarkably limited to CD4^+^CD38^+^ T cells, indicating that they may contribute to disease chronicity and persistent inflammation.

While the current paradigm in CD4 T cell biology considers chronic infections to be a trigger for anergy and T cell hyporesponsiveness, persistent and low-grade exposure to antigens is, in fact, essential for maintenance of polyfunctional T cells^23,26^, which may explain the heightened basal response in AD patients. Thus, multiparametric flow cytometry studies reveal a large phenotypic and functional heterogeneity in T cell responses, highlighting the importance of assessing multiple attributes of T cell function to predict the outcome of infections, the course of immune diseases and the success of vaccination protocols^23,44^.

Novel therapeutic options in AD aiming immune system via microbiome modulation, should focus on host-pathogen interaction, associating specific effector T-cell subsets with specific neutralizing anti-toxins antibodies. Future therapeutic targets strategies for AD should consider the interaction between host and pathogen, by focusing on cytokine release by activated effector T cells population-level responses to pathogens such as staphylococcal enterotoxins^19,45,46^.

In conclusion, our findings help to corroborate the pathogenic role of staphylococcal enterotoxins in modulating the cytokine release by activated effector T cells. Altered gene expression, such as *EGR2* induction, and impaired polyfunctional response may contribute to the negative regulation of these activated CD4^+^ T cells in adults with AD. Our data reveal fundamental insights into how individual cells dynamically modulate intercellular signals to affect population-level responses to pathological conditions or clinical interventions.

## Methods

### Subjects

Fifteen AD patients (aged between 20–43 years; mean age: 28.53 ± 7.67; 9 males and 6 females), and 10 healthy non-AD volunteers (aged between 20–41 years; mean age: 29.7 ± 6.43; 7 males and 3 females) were included in this study. AD was diagnosed agreeing with the Hanifin & Rajka criteria^47^. Disease severity was assessed by the EASI (Eczema Area and Severity Index)^48^, and AD patients were categorized as mild (n = 2), moderate (n = 8), or severe (n = 5). IgE serum levels varied from 2,680 to 119,000 IU/mL (average of 25,437). None of the patients were under immunosuppressants or oral steroids treatment. This study was according to the Ethics Committee of the University of Sao Paulo School of Medicine, and informed consent was obtained from all subjects. All methods were achieved in accordance with the pertinent guidelines and regulations of this institution. Demographic information is shown in Table 1.

**Table 1:** Demographic data of adults with AD.

Identification	Gender	Age (years)	EASI	IgE (IU/mL)	Eosinophils %
AD1	M	30	48.4	18,300	6.6
AD2	M	29	40.2	16,700	19.4
AD3	F	39	31.6	6,120	5.2
AD4	M	28	40.2	16,700	6.4
AD5	M	24	48.9	3,120	10.1
AD6	F	37	48.4	59,900	5.1
AD7	M	43	18.4	28,300	12.7
AD8	F	24	49	21,200	5.2
AD9	M	24	50	46,300	22
AD10	M	39	35.4	5,080	6.7
AD11	F	20	34.8	119,000	5.5
AD12	M	20	14.4	4,910	7.6
AD13	F	21	48.2	54,200	9.9
AD14	F	29	11.2	4,070	14.2
AD15	M	21	38.5	2,870	6.8
HC (n = 10)	7 F/3 M	29.7 (24–41)	NA	<100	<5

AD = atopic dermatitis; HC = healthy controls; M = male; F = female; EASI = Eczema Area and Severity Index; NA = not applicable.

### CD4^+^ T cell sorting

PBMCs (50 × 10^6^/mL) were incubated with anti-CD3-Horizon BV605 (10 μL – Clone: SK7/BD Bioscience), anti-CD4-Horizon V450 (10 μL – Clone: RPA-T4/BD) and anti-CD8-PerCP-Cy 5.5 (20 μL – Clone: RPA-T8/BD) for 30 minutes at 4 °C. The cells were washed in 1x sterile PBS (1 mL) and resuspended in RPMI 1640 medium (1 mL, Gibco) supplemented with 10% of human AB serum (Sigma). Cell sorting was performed with a FACSAria III cell sorter (BD Biosciences), and a 99% pure population of 2.5 × 10^6^ CD3^+^CD4^+^CD8^−^ cells (CD4^+^ T cells) was collected.

### T cell anergy PCR array

FACS-sorted CD4^+^ T cells (2 × 10^6^/mL) were assessed under unstimulated conditions and after staphylococcal enterotoxin A (SEA) stimulation (0.1 μg/mL) for 3 hours, at 37 °C and 5% CO_2_. Total cellular RNA was extracted from CD4^+^ T cells using an RNeasy Plus Mini Kit (Qiagen, Valencia, CA, USA). The first strand of cDNA was synthesized using the RT^2^ First Strand kit and PreAMP kit (Qiagen) according to the manufacturer’s instructions, and the final cDNA product was used for the RT^2^ Profiler PCR Array using SYBR Green-based real-time PCR according to the manufacturer’s protocol (PAHS-074Z Array, Qiagen). The array consisted of a panel of 84 genes relevant to T cell anergy and immune tolerance (Fig. 1a) plus five reference genes (*B2M*, *HPRT1*, *RPL13A*, *GAPDH* and *ACTB*), a genomic DNA control, three reverse transcription controls and three PCR quality controls. Only samples passing the PCR array run quality control, which confirmed the absence of genomic DNA contamination and proper amplification of the reverse transcription controls and the positive PCR controls, were further evaluated. For the analysis, the Ct values were normalized with the geometric average of the Ct of the reference genes (ΔCt), and the relative expression was calculated based on the median ΔCt values of the healthy control group in the basal condition, according to the Livak^49^ method as adjusted by Liu & Saint^50^. Data were presented as the log_2_ of the relative expression values in a heatmap generated by the R software (v3.3.2. for Windows) with the following packages: gplots and RColorBrewer. Volcano plots were created using the software “R” for Mac OS X GUI (v3.6.0; package “ggplot2”) based on the fold change and *p*-values between atopic dermatitis patients and health controls.

### RT-qPCR

The final cDNA product was generated from FACS-sorted CD4^+^ T cells under unstimulated conditions and after SEA stimulation. Reverse transcription was performed using an iSCRIPT Reverse Transcriptase kit (Bio-Rad, Hercules, California, USA).

Reverse transcription-quantitative polymerase chain reaction (RT-qPCR) was performed in an Applied Biosystems 7500 system using specific primers and SYBR Green (Applied Biosystems, Carlsbad, CA, USA), as described by Pereira *et al*.^51^. Primers for RT-qPCR (Life Technologies), were only used if their efficiency achieved 100 ± 10%. Adjustments were made for primer efficiency. The specificity of the reaction was assessed by dissociation curve. The glyceraldehyde-3-phosphate dehydrogenase (*GAPDH*) mRNA levels of the samples in the same plate were analyzed to normalize the mRNA contents among the tested samples. The cycling protocol comprised of 10 minutes at 95 °C, followed by 40 cycles of 15 seconds at 95 °C, and 60 seconds at 60 °C. The amplification results were evaluated using Sequence Detection System (SDS) software (Applied Biosystems). ΔCt was calculated as the difference between the Ct value of a gene and the geometric average of the Ct values of the reference genes. Log-fold differences in expression were reported using the 2−ΔΔCt or 2−ΔCt as described by Livak^49^.

The following primers were used: *EGR2* gene: forward primer (5′-ACGTCGGTGACCATCTTTCC-3′) and reverse primer (5′-GTTGATCATGCCATCTCCGG-3′); *IL13* gene: forward primer (5′- GCAATGGCAGCATGGTATGG-3′) and reverse primer (5′-CTGCACAGTACATGCCAGCT-3′); and *GAPDH* gene: forward primer (5′-GAAGGTGAAGGTCGGAGT-3′) and reverse primer (5′- GAAGATGGTGATGGGATTTC-3′).

### Flow cytometry

Peripheral blood mononuclear cells (PBMCs) from heparinized venous blood were isolated with a Ficoll-Hypaque (GE Healthcare, Uppsala, Sweden) gradient and resuspended in RPMI 1640 medium supplemented with gentamicin (40 µg/mL) and 10% pooled AB normal human serum (Sigma-Aldrich, St Louis, MO, USA). PBMCs (2 × 10^6^/well) were cultivated in 96-well microplates (Costar) at 37 °C and 5% CO_2_ in the presence of SEA (0.04 µg/mL; Sigma) for 6 hours. Brefeldin A (10 µg/mL; Sigma) was added 2 hours after the culture was initiated. Next, the cells were stained with a PE-Texas Red LIVE/DEAD viability marker (Invitrogen, Carlsbad, CA, USA) for 30 minutes at room temperature. Cells were then incubated with Cytofix/Cytoperm (BD Biosciences, San Jose, CA, USA) and stained with the following antibodies: anti-CD3-Qdot 605 (Invitrogen), anti-CD4-Horizon V500 (BD), anti-CD8-PerCP-Cy5.5 (BD), anti-TNF-PE-Cy7 (BD), anti-CD38-Alexa 700 (BD), anti-IL-17A-Alexa 488 (eBioscience, San Diego, CA, USA), anti-IL-22-PE (eBioscience), anti-MIP-1β-APC-H7 (BD) and anti-IFN-γ-Horizon V450 (BD Biosciences). Cells were then fixed with 1% paraformaldehyde, and 400,000 events were acquired on an LSR Fortessa flow cytometer (BD Biosciences). Fluorescence minus one (FMO) controls were performed for all antibody panels to check for proper compensation and to define positive signals. Data analysis was performed using FlowJo v10 software (Tree Star, Ashland, OR, USA). After gates were created to define each individual function, we used the Boolean gate platform of the FlowJo software to create an array of possible cytokine combinations. Principal Component Analysis (PCA) of the polyfunctional profile was made in R software (v3.5.1, for Windows) with the following packages: ggplot2 and ggbiplot.

### Statistical analysis

The Mann–Whitney test or Kruskal–Wallis test with Dunn’s post hoc test was utilized to compare 2 or 3 sets of data, respectively. Correlations were established using the Spearman nonparametric correlation test. Differences between groups were considered statistically significant when p < 0.05.

## Supplementary information

Dataset 1
