# Supplementary material for: Staphylococcal enterotoxins modulate the effector CD4+ T cell response by reshaping the gene expression profile in adults with atopic dermatitis
Source: Sci Rep. 2019 Sep 11;9:13082. doi: 10.1038/s41598-019-49421-5 (PMC6739319; doi:10.1038/s41598-019-49421-5)
Supplement: Supplementary file 1 — Dataset 1 [file 41598_2019_49421_MOESM1_ESM.pdf]

Title: Staphylococcal enterotoxins modulate the effector $CD4^+$ T cell response by reshaping the gene expression profile in adults with atopic dermatitis

Authors: Raquel Leao Orfali*, Fabio Seiti Yamada Yoshikawa, Luanda Mara da Silva Oliveira, Natalli Zanete Pereira, Josenilson Feitosa de Lima, Yasmim Álefe Leuzzi Ramos, Alberto José da Silva Duarte, Maria Notomi Sato[+], Valeria Aoki[+].

Department of Dermatology, Laboratory of Dermatology and Immunodeficiencies (LIM-56), Faculdade de Medicina FMUSP, Universidade de Sao Paulo, Sao Paulo, SP, BR

[+]These authors shared the mentorship, critical revision and supervision of this study.

*Corresponding author: Raquel Leao Orfali, MD, PhD.
Department of Dermatology, Faculdade de Medicina FMUSP, Universidade de Sao Paulo
Address: Av. Dr. Eneas de Carvalho Aguiar, 255, 3o. andar ICHC, Sala 3016, Cerqueira Cesar, Sao Paulo, SP, BR. CEP-05403-002. Email: raquelleao@hotmail.com

Supplementary figures

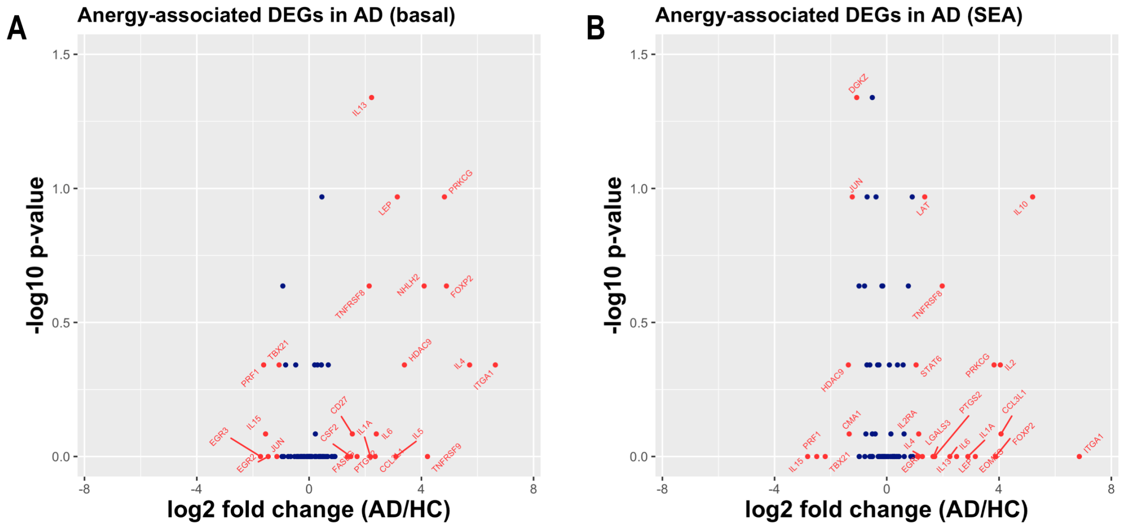

Supplementary Figure S1. Anergy-associated differentially expressed genes (DEGs) in atopic dermatitis. Volcano plots show DEGs between atopic dermatitis, *AD*, and health controls, *HC*, in basal condition (A) and after SEA stimulation (B). Positive values in *x*-axis indicate genes up-regulated in AD group whereas negative values point to genes up-regulated in HC group. Genes whose absolute fold change values (log2) were ≥ 1 were highlighted in red and labeled.

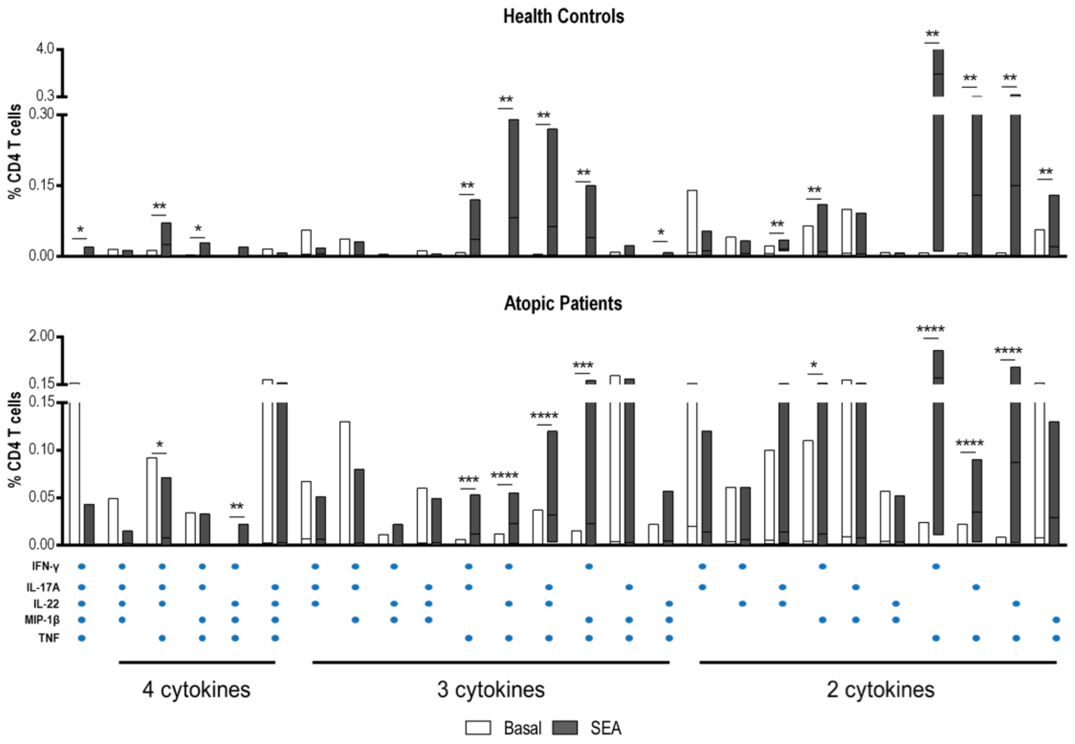

Supplementary Figure S2: Altered cytokine polyfunctional response in CD4⁺ T cells after SEA stimulation in AD. Functional analysis of the frequency of the CD4⁺ T cells from atopic dermatitis patients (AD, n = 15) and healthy controls (HC, n = 10) that were positive for IL-17A, IL-22, IFN-γ, MIP-1β or TNF without (Basal) and after SEA stimulation was performed by multiparametric flow cytometry. Lines represent the median percentage of population positivity for each cytokine combination. *p<0.05, **p<0.01, ***p<0.001, ****p<0.0001.

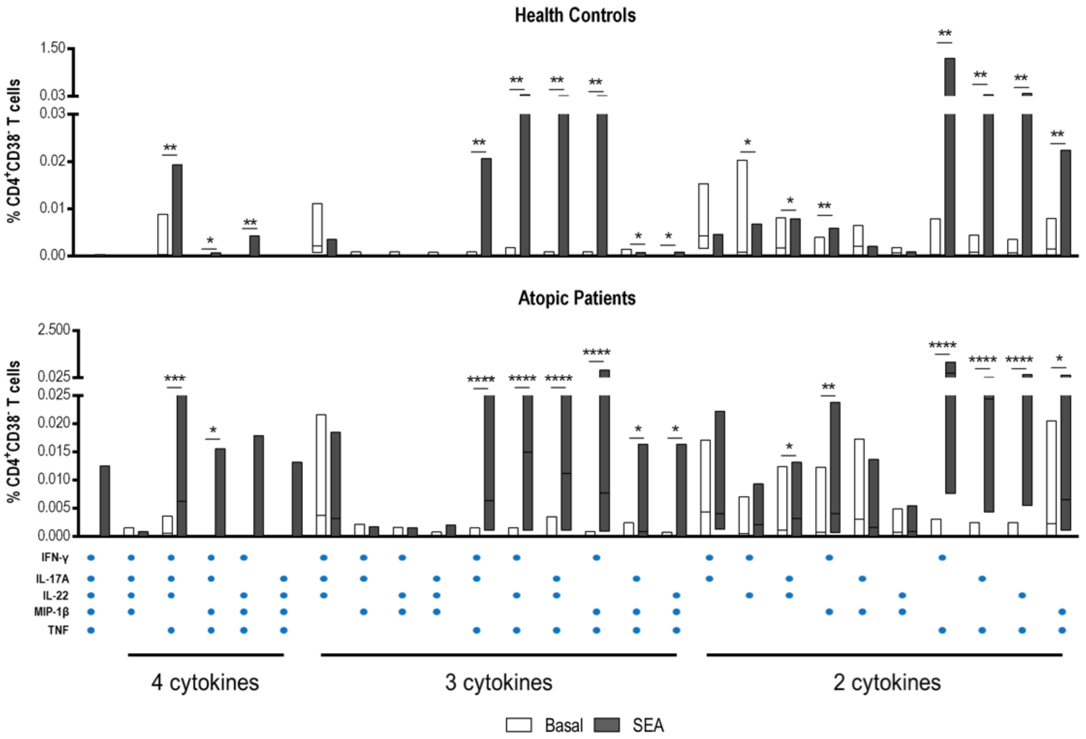

Supplementary Figure S3: Cytokine polyfunctional response in $CD4^{+}CD38^{-}$ T cells after SEA stimulation in AD. Frequencies of polyfunctional T cells at baseline and after SEA stimulation from HC (n = 10) compared to AD patients (n = 15) were performed by multiparametric flow cytometry. Lines represent the median secretion of each cytokine combination. *p<0.05, **p<0.01, ***p<0.001, ****p<0.0001.

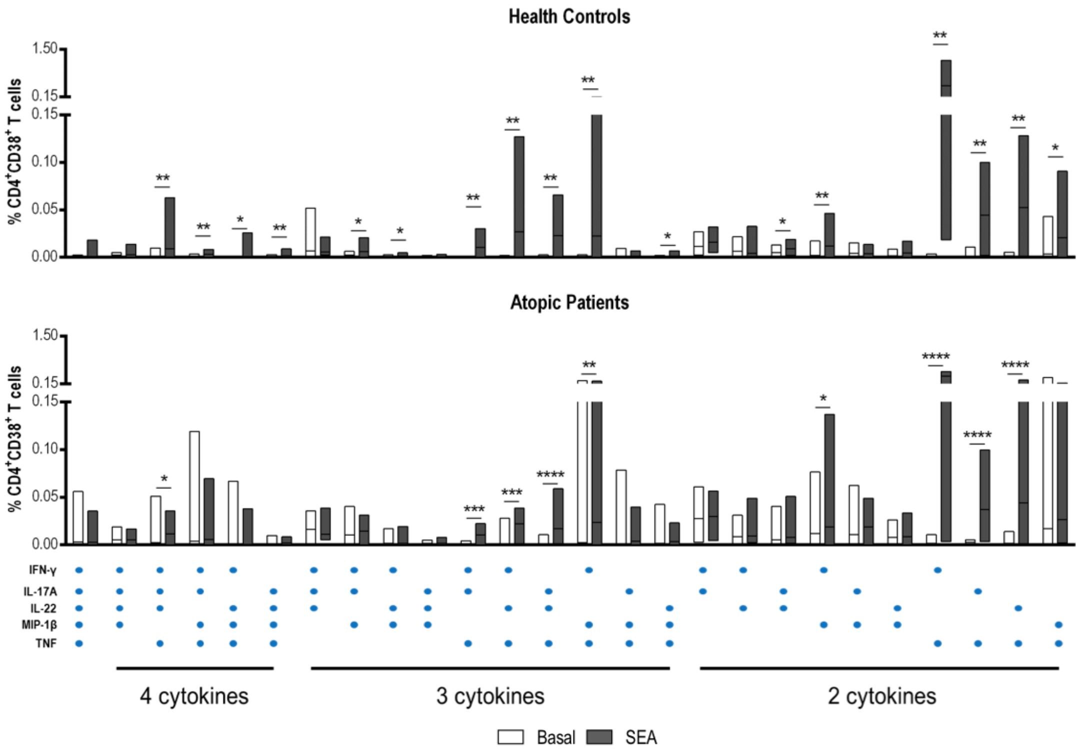

Supplementary Figure S4: Altered cytokine polyfunctional response in $CD4^{+}CD38^{+}$ T cells is associated with a dysfunctional response to SEA in AD patients. Frequencies of polyfunctional T cells at baseline and after SEA stimulation from HC (n = 10) compared to AD patients (n = 15) were performed by multiparametric flow cytometry. Lines represent the median secretion of each cytokine combination. *p<0.05, **p<0.01, ***p<0.001, ****p<0.0001.
